# Do trajectories of economic, work- and health-related disadvantages explain child welfare clients’ increased mortality risk? A prospective cohort study

**DOI:** 10.1186/s12889-019-6752-y

**Published:** 2019-04-18

**Authors:** Ylva B. Almquist, Lars Brännström

**Affiliations:** 1 Department of Public Health Sciences, Centre for Health Equity Studies (CHESS), Stockholm University, SE-106 91 Stockholm, Sweden; 2 Department of Social Work, Stockholm University, SE-106 91 Stockholm, Sweden

**Keywords:** Cohort, Life-course, Longitudinal, Out-of-home care, Mortality, Socioeconomic status, Sweden

## Abstract

**Background:**

Past research has shown that individuals who have had experiences of out-of-home care (OHC) in childhood have increased risks of premature mortality. Prior studies also suggest that these individuals are more likely to follow long-term trajectories that are characterised by economic, work-, and health-related disadvantages, compared to majority population peers. Yet, we do not know the extent to which such trajectories may explain their elevated mortality risks. The aim of this study is therefore to examine whether trajectories of economic, work-, and health-related disadvantages in midlife mediate the association between OHC experience in childhood and subsequent all-cause mortality.

**Methods:**

Utilising longitudinal Swedish data from a 1953 cohort (*n* = 14,294), followed from birth up until 2008 (age 55), this study applies gender-specific logistic regression analysis to analyse the association between OHC experience in childhood (ages 0–19; 1953–1972) and all-cause mortality (ages 47–55; 2000–2008). A decomposition method developed for non-linear regression models is used to estimate mediation by trajectories of economic, work-, and health-related disadvantages (ages 39–46; 1992–1999), as indicated by social welfare receipt, unemployment, and mental health problems. To account for selection processes underlying placement in OHC, an alternative comparison group of children who were investigated by the child welfare committee but not placed, is included.

**Results:**

The results confirm that individuals with experience of OHC have more than a two-fold increased risk of all-cause mortality, for men (OR: 2.10, 95% CI: 1.42–3.11) and women (OR: 2.23, 95% CI: 1.39–3.59) alike. Approximately one-third (31.1%) of the association among men, and one-fourth (27.4%) of the association among women, is mediated by the long-term trajectories of economic, work-, and health-related disadvantages. The group who were investigated but not placed shows similar, yet overall weaker, associations.

**Conclusions:**

Individuals who come to the attention of the child welfare services, regardless of whether they are placed in out-of-home care or not, continue to be at risk of adverse outcomes across the life course. Preventing them from following trajectories of economic, work-, and health-related disadvantages could potentially reduce their risk of premature death.

## Background

The link between child maltreatment and health inequalities remains an area of concern for researchers, policymakers and service providers, and presents an enduring threat to public health [1]. Child welfare interventions such as placement in out-of-home care (OHC; foster family or residential care) are intended to provide a temporary haven for children who are maltreated (abused and/or neglected) by their parents or adolescents with significant antisocial behaviour problems. In Sweden, the context of the current study, a core aim of OHC placement is also to provide the child with better opportunities for development [2]. Yet, prior research has consistently identified children with experience of OHC as a high-risk group for premature mortality [3–9], the perhaps most readable indicator of health inequality.

How to understand this seemingly robust association between childhood experience of OHC and life expectancy? The ‘chain-of-risk’ model [10] maintains that health risks are linked, meaning that one exposure leads to another, and then another after that. Accordingly, the reason for being placed in OHC could be seen as a fundamental exposure, setting off an adverse chain of events, which ultimately leads up to poor health in adulthood. Reasons for placement are – at least in early and middle childhood – primarily related to adverse family circumstances rather than to the child’s own behaviour. Such circumstances include parents’ behavioural and mental problems but also socioeconomic factors. For example, also after taking parental addiction and psychiatric problems into account, children living with single mothers and whose mothers have low education and receive social assistance, have higher risks of entering OHC [11]. From a vast literature focusing on social determinants of health, we know that these aspects are also risk factors for poor health-related outcomes in adulthood [12].

A chain-of-risk model does, however, suggest that single exposures restricted to a certain stage of life do not sufficiently address the complexity of the life course. Previous research has shown that children who are placed in OHC have increased risks of following disadvantaged socioeconomic and health-related trajectories across adulthood [13] and that they are also more likely to experience a greater accumulation of such adversities over time [14]. Moreover, studies of such life-long trajectories in the general population show that individuals with sustained disadvantage have higher risks of mortality, both from all causes and from specific causes such as cardiovascular diseases, cancers, and external causes [15, 16]. We still, however, have limited knowledge of the role that long-term trajectories of economic, work-, and health-related disadvantages may play for mortality among children with experience of placement in OHC.

Using prospective data from a cohort of more than 14,000 individuals who were born in 1953 and lived in Metropolitan Stockholm (the capital region of Sweden) at age 10, of which nearly 9% have experience of OHC during their upbringing, the aim of the current study is to further our understanding about how trajectories of economic, work-, and health-related disadvantages are related to the increased risk for premature mortality in individuals with a childhood history of involvement in the child welfare system. We apply a decomposition method developed for non-linear regression models [17] to assess whether and to what extent these trajectories (ages 39–46) mediate the association between childhood experience of OHC (ages 0–19) and subsequent mortality (ages 47–55). Moreover, we also include a comparison group consisting of individuals that were investigated by the child welfare committee, but were never placed in OHC. This contrast may to some extent account for the selective processes that lead to placement.

## Methods

The data material used was the Stockholm Birth Cohort Study (SBC) which is defined as all individuals who were born in 1953, resided in the greater Stockholm metropolitan area in 1963, and were still alive and resident in Sweden in 1980 and/or 1990 (*n* = 14,294) [18, 19].

We derived information about OHC (ages 0–19; 1953–1972) from manually collected data from the Social Registers of municipalities in the Stockholm region. Placement in OHC was defined as at least one record of being placed in either family foster care or residential care due to family-related problems or own behavioural problems. The records in the original data had been collapsed into three time periods: 1953–1969 (ages 0–6), 1960–1965 (ages 7–12, and 1966–1972 (ages 13–19). It can be noted that among those who were placed, 83% had their first placement in ages 0–6, 12% in ages 7–12, and 5% in ages 13–19. Our data also identify children who had come to the attention of the child welfare committee but had not been placed. For the analysis, three groups were created: ‘Neither investigated nor placed’, ‘Investigated but not placed’, and ‘Placed’. Table 4 in the Appendix shows the distribution of family-related characteristics across these groups: children in the category ‘Placed’ were much more likely to come from manual class backgrounds and have parents who received social welfare or had mental health problems, compared to children in the category ‘Neither investigated nor placed’. The group of ‘Investigated but not placed’ placed itself somewhere in between.

Trajectories of economic, work-, and health-related disadvantages (ages 39–46; 1992–1999) were estimated in a previous study based on the same data material, where they were examined in relation to subsequent mortality risks [15]. The trajectories were based on annual information about social welfare receipt (S), unemployment (U), and mental health problems (M). Of these indicators, the two first were gathered from the Longitudinal Integration Database for Health Insurance and Labour Market Studies (LISA) whereas the third was based on cases of in-patient care drawn from the National Patient Register. By forming a binary indicator (no/yes) for each disadvantage, an eight-category measure was created to reflect all possible combinations in each given year. Following this, sequence analysis and hierarchical cluster analysis were applied (for men and women separately), resulting in seven clusters of economic, work-, and health-related trajectories; these are further described in Table 1.

**Table 1:** Descriptive statistics of the study variables (*n* = 13,468)

	Men *n* = 6849					Women *n* = 6619				
	n	%	% among Investigated but not placed	% among Placed	% Deaths	n	%	% among Investigated but not placed	% among Placed	% Deaths
Experience of OHC (1953–1972)										
Not investigated nor placed	5205	76.0				5714	86.3			
Investigated but not placed	1008	14.7				348	5.3			
Placed	636	9.3				557	8.4			
Trajectories of economic, work-, and health-related disadvantages: Men (1992–1999)										
1. No S, U, or M	5207	76.0	66.9	59.3	2.3					
2. U, early 1990s	469	6.9	7.4	7.2	2.8					
3. S and/or U	275	4.0	6.1	6.6	5.1					
4. S and/or U, early 1990s	262	3.8	5.9	7.4	4.6					
5. S and/or U, mid-late 1990s	259	3.8	5.0	4.7	4.3					
6. S, U, and/or M, high levels	234	3.4	6.1	11.8	12.4					
7. U, late 1990s	143	2.1	2.8	3.0	4.9					
Trajectories of economic, work-, and health-related disadvantages: Women (1992–1999)										
1. No S, U, or M						4092	61.9	43.7	40.4	1.4
2. S, U, and/or M, low levels						1022	15.4	21.3	26.6	1.8
3. U, early 1990s						553	8.4	12.1	7.7	2.5
4. U						304	4.6	6.9	5.4	2.0
5. U, mid-1990s						274	4.1	4.6	3.2	1.5
6. S, U, and/or M, high levels						211	3.2	5.5	9.3	7.6
7. S, high levels, with some U and/or M						161	2.4	6.0	7.4	8.7
All-cause mortality (2000–2008)										
No	6644	97.0	95.8	94.5		6488	98.0	96.8	95.9	
Yes	205	3.0	4.2	5.5		131	2.0	3.2	4.1	

*S* Social welfare receipt, *U* Unemployment, *M* Mental health problems

Information about (premature) mortality was taken from the Causes of Death Register (ages 47–55; 2000–2008). Again, this follow-up period was selected to correspond to an earlier study on the mortality risks associated with trajectories of economic, work-, and health-related disadvantages [15]. The most frequent causes of death were cancer, alcohol and drug use disorders, circulatory disease, injuries, and suicide. However, since the number of deaths was too small to examine specific causes, all-cause mortality was used as the outcome in this study.

### Statistical analysis

Individuals who died before the mortality follow-up (*n* = 341), as well as those who had missing information for the sequences (*n* = 485) were excluded from the analysis. This reduced the analytical sample from 14,294 to 13,468 individuals. We subsequently applied gender-specific logistic regression models with KHB-estimation (KHB is short for Karlson, Holm, and Breen) to analyse the association between OHC experience in childhood and mortality, as well as the mediating role of the trajectories of economic, work-, and health-related disadvantages. With KHB, it was possible to decompose the total effect of OHC experience into a direct (i.e. not mediated by the trajectories) and an indirect (i.e. mediated by the trajectories) part. Since only one model is derived, the analysis provides estimates that are free from rescaling bias, a problem well-known for logistic regression models [17]. The analysis was performed in two steps: in the first, individuals who had neither been investigated not placed in OHC constituted the reference group and, in the second step, the reference group was changed to those who had been investigated but not placed.

It would have been preferable to apply hazard regression analysis, such as Cox, given the fact that the outcome was measured over several years. However, KHB-estimation is not available for Cox models. A sensitivity analysis (data not presented), without KHB-estimation, nevertheless showed that the odds ratios derived from the logistic regression analysis were not substantially different from the hazard ratios derived from Cox regression analysis.

## Results

Table 2 demonstrates the associations between experience of OHC and all-cause mortality among men, based on logistic regression models with KHB-estimation. The upper-most part of the table shows the mortality risks for men who were placed and investigated but not placed, respectively, in comparison to those who were neither investigated nor placed in OHC. To start with, those who were investigated but not placed have a higher, statistically significant, risk of all-cause mortality (OR: 1.64, 95% CI: 1.14–2.34). The estimate is even larger among men with experience of being placed in OHC, who display more than a two-fold increased risk (OR: 2.10, 95% CI: 1.42–3.11). The subsequent model includes adjustment for trajectories of economic, work-, and health-related disadvantages, leading to a reduction of the associations corresponding to 25% for those who were investigated but not placed, and 31.1% among those who were placed. In the lower-most part of the table, the reference group has been alternated and instead includes those who were investigated but not placed. There is still a statistically significant difference in all-cause mortality between this group and those who were neither investigated nor placed (OR: 0.61, 95% CI: 0.43–0.88), but not when the individuals only subjected to investigation are compared to those who were placed in OHC (OR: 1.28, 95% CI: 0.81–2.05). Nevertheless, when comparing the two latter categories, the percentage of the association explained by the trajectories of economic, work, and health-related disadvantages amounts to 42.6.

**Table 2:** Associations between experience of OHC and all-cause mortality among men, based on logistic regression analysis with KHB-estimation (*n* = 6849). Statistically significant estimates (*p* < 0.05) are presented in bold

	All-cause mortality		
	Men (*n* = 6849)		
	Unadjusted	Adjusted^a^	% explained
	OR (95% CI)	OR (95% CI)	
Experience of OHC			
Neither investigated nor placed (ref.)	1.00	1.00	
Investigated but not placed	**1.64** (1.14–2.34)	**1.45** (1.01–2.08)	25.0
Placed	**2.10** (1.42–3.11)	**1.67** (1.12–2.50)	31.1
Experience of OHC			
Neither investigated nor placed	**0.61** (0.43–0.88)	**0.69** (0.48–0.99)	25.0
Investigated but not placed (ref.)	1.00	1.00	
Placed	1.28 (0.81–2.05)	1.15 (0.72–1.84)	42.6

^a^Adjusted for trajectories of economic, work-, and health-related disadvantages

In Table 3, the corresponding results for women are shown. Starting with the upper-most part of the table, there is no statistically significant difference in all-cause mortality between those who were investigated but not placed and women who were neither investigated nor placed (OR: 1.75, 95% CI: 0.92–3.32). However, similar to men, the results indicate more than a two-fold increased risk for individuals with experience of being placed in OHC (OR: 2.23, 95% CI: 1.39–3.59). In the adjusted model, the trajectories of economic, work-, and health-related disadvantages explain 28.1 and 27.4%, respectively for the groups. Next, for the lower-most part of Table 3, the results show that there are no statistically significant differences comparing individuals who were investigated but not placed with those who were neither investigated nor placed (OR: 0.57, 95% CI: 0.31–1.08), or those who were placed in OHC (OR: 1.28, 95% CI: 0.61–2.67). The percentages of the associations that are explained by the trajectories are estimated to 28.1 and 25.8, respectively.

**Table 3:** Associations between experience of OHC and all-cause mortality among women, based on logistic regression analysis with KHB-estimation (*n* = 6619). Statistically significant estimates (*p* < 0.05) are presented in bold

	All-cause mortality		
	Women (*n* = 6619)		
	Unadjusted	Adjusted^a^	% explained
	OR (95% CI)	OR (95% CI)	
Experience of OHC			
Neither investigated nor placed (ref.)	1.00	1.00	
Investigated but not placed	1.75 (0.92–3.32)	1.50 (0.78–2.86)	28.1
Placed	**2.23** (1.39–3.59)	**1.79** (1.10–2.92)	27.4
Experience of OHC			
Neither investigated nor placed	0.57 (0.31–1.08)	0.67 (0.35–1.28)	28.1
Investigated but not placed (ref.)	1.00	1.00	
Placed	1.28 (0.61–2.67)	1.20 (0.57–2.51)	25.8

^a^Adjusted for trajectories of economic, work-, and health-related disadvantages

## Discussion

The aim of this study was to investigate the associations between childhood experience of OHC, long-term trajectories of economic, work-, and health-related disadvantages, and subsequent mortality. First of all, our results show that OHC experience is associated with men’s and women’s subsequent risks of premature death. The strength of this association is weaker in comparison to previous studies of OHC and mortality based on the same cohort [4]. A probable reason is that the follow-up of mortality in the current study is much more limited (spanning the years 2000–2008 instead of 1973–2008). Children with experience of OHC do not only have increased risks of premature death, they also tend to die earlier than their majority population peers. Thus, while this may give the appearance of decreasing inequalities in mortality in later life, it is most likely a matter of non-random selection processes [20].

A previous study based on sequence analysis found that children with OHC experience are more likely to follow adverse socioeconomic and health-related trajectories, particularly trajectories characterised by greater complexity and longer duration of disadvantage [13]. That study built the trajectories on four indicators – low educational attainment, social welfare receipt, unemployment, and mental health problems – and the follow-up stretched across ages 39–55. Since the current study measured all-cause mortality succeeding the trajectories, our trajectories spanned a shorter time period (ages 39–46). For this purpose, we utilised the results from another study that applied sequence analysis to examine all-cause mortality [15]. Our trajectories are consequently based on three indicators of adversity in midlife: social welfare receipt, unemployment, and mental health problems. Yet, the descriptive results of the present investigation show the similar patterns as in previously mentioned study: compared to majority population peers, those who were placed in OHC are much more likely to follow trajectories that are characterised by two or three simultaneous disadvantages across the entire measurement period.

The key finding in this study is that the trajectories of economic, work-, and health-related disadvantages account for approximately one-third (31.1%) of male child welfare clients’, and one-fourth (27.4%) of female child welfare clients’ increased mortality risks in adulthood. This may be considered a substantial part of the association, particularly since the trajectories only capture three aspects of disadvantage over the course of 8 years. It is highly likely that these percentages would increase if we would have been able to measure them in more detail and for a longer period of time. Concerning the specific indicators of economic, work-, and health-related disadvantages used in the current study, some limitations should be noted. For example, our measure of mental health problems is based on in-patient care which only reflects the most severe cases. In order to capture a more comprehensive picture, it would have been preferable to use out-patient care.

Either way, the results indicate that children who are placed in OHC tend to embark on an adverse journey through life, encountering subsequent disadvantages in terms of economic difficulties, poor labour market attachment, and mental health problems. Moreover, these disadvantages often go hand-in-hand, piling up across the life course. For some individuals, such cumulative processes ultimately lead to premature death. Future studies should aim at explaining the remaining part of the association by considering other indicators of disadvantage, measured from adolescence and onwards, such as behavioural problems, poor housing, somatic ill-health, low general well-being, early childbearing and family disruption, and poor educational attainment. In particular, past research has identified educational failure as a fundamental explanation for the adverse long-term outcomes of child welfare clients [9, 21–23].

The current study also included an alternative comparison group in the analysis: individuals who came to the attention of the child welfare committee but were not placed. They too showed increased risks of mortality compared to their majority population peers, whereas there was no statistically significant difference in mortality in relation to individuals who were placed in OHC. This could be taken as a confirmation of selective processes – or ‘confounding by indication’ [24] – underlying the link between OHC and mortality. However, previous studies on the same cohort have established that those with OHC experience indeed have significantly higher risks. We argue, once again, that this might be due to the restricted follow-up of mortality, which also affects the power of the analysis and thus the ability to detect statistically significant differences. However, if children who are placed in OHC die *earlier* than those who are investigated but not placed, it could be the case that the differences between the two groups are actually smaller among those who survived until the start of the follow-up. This argument is to some extent supported by the descriptive statistics, showing that there were no large differences between the two groups regarding the trajectories of economic, work-, and health-related disadvantages, which could also explain why the percentage mediated by the trajectories was comparable between the groups.

The Swedish birth cohort analysed in the current study grew up under different conditions compared to more recent cohorts. In the 1950s and 1960s, the Swedish welfare state was developing rapidly, with improved living conditions for its citizens. Moreover, the ethnic composition of the population was highly homogenous. This in striking contrast to Swedish society of today, in which ethical background is of great relevance for the analysis of OHC. Apart from ethnicity, the cohort members who entered OHC also differed in other respects from children who are removed from their parents today. For example, children in our cohort were more likely to be placed due to presumed maternal immaturity, poor housing conditions, household poverty, and single motherhood, or own behavioural problems [25], whereas placement in OHC today is more likely to result from child abuse and neglect, maternal drug misuse, and domestic violence. It is therefore reasonable to expect children and young people who exit OHC today to experience more trauma before entering care, compared to our cohort. Yet, there are also studies suggesting that a significant part of the child welfare population in the years between 1950 and 1980 experienced inferior and hostile care [26]. Regardless of this, however, we cannot avoid using historical data if we wish to explore the pathways through which exposure to OHC may be related to later death.

## Conclusions

The findings of the current study suggest that individuals who come to the attention of the child welfare services, regardless of whether they are placed in out-of-home care or not, continue to be at risk of adverse outcomes across the life course. Preventing them from following trajectories of economic, work-, and health-related disadvantages could potentially reduce their risk of premature death. In contrast to many other Western countries, Sweden lacks systematic aftercare support for children aging out of OHC [27]. Since such support has been linked to improved life-course outcomes [28], development and implementation of aftercare services seem like a viable prevention path.

## Appendix

**Table 4:** Family-related characteristics and experience of OHC (*n* = 13,468)

	Neither investigated nor placed	Investigated but not placed	Placed
	%	%	%
Household occupational class^a^			
Upper/upper middle class	14.8	7.5	3.2
Middle class	38.5	31.3	28.7
Working class	46.6	58.4	64.0
Unclassified	3.1	2.9	4.2
Social welfare receipt^b^			
Number of years of receipt (mean)	0.56	1.86	4.06
Parental mental health problems^c^			
No	86.8	87.5	71.6
Yes	3.2	12.5	28.4
*Total*	*100*	*100*	*100*

^a^Refers to the occupation of the head of the household, derived from occupational registers (1953)

^b^Refers to number of years that the family received social welfare, derived from the Social Register (1953–1972)

^c^Based on records of fathers’/mothers’ mental health problems, derived from the Social Register (1953–1972)

## Abbreviations

CI: Confidence intervals
KHB: Karlson-Holm-Breen
LISA: Longitudinal Integration Database for Health Insurance and Labour Market Studies
OHC: Out-of-home care
OR: Odds ratio
SBC: The Stockholm Birth Cohort Study
